# High-throughput sequencing of RNAs isolated by cross-linking immunoprecipitation (HITS-CLIP) reveals Argonaute-associated microRNAs and targets in *Schistosoma japonicum*

**DOI:** 10.1186/s13071-015-1203-9

**Published:** 2015-11-14

**Authors:** Jing Zhao, Rong Luo, Xindong Xu, Ying Zou, Qingfeng Zhang, Weiqing Pan

**Affiliations:** Institute for Infectious Diseases and Vaccine Development, Tongji University School of Medicine, Shanghai, China; Department of Tropical Infectious Diseases, Second Military Medical University, Shanghai, China

**Keywords:** *Schistosoma japonicum*, HITS-CLIP, microRNA, Argonaute, Target sites

## Abstract

**Background:**

Schistosomiasis, caused by *schistosomes*, is one of the most prevalent and serious parasitic diseases in tropical and subtropical countries. This pathogen has a complex life cycle and harbors a unique repertoire of genes expressed at different life-stages. Understanding the gene regulation of *schistosomes* will contribute to identification of novel drug targets and vaccine candidates. Some conserved and novel microRNAs (miRNAs) have been identified in *schistosomes* as key transcriptional and post-transcriptional regulators in the past few years; however, little is known about their specific targets.

**Methods:**

High-throughput sequencing of RNAs isolated by cross-linking immunoprecipitation (HITS-CLIP) was used to covalently crosslink native Argonaute protein-RNA complexes in *Schistosoma japonicum.* An antibody against *S.japonicum* Argonaute proteins, was generated and used for immunoprecipitation of the crosslinked SjAgo-RNA complex from soluble adult worm extract. Small RNAs, including miRNAs and their target mRNAs associated with the native SjAgo in adult parasites, were enriched and extracted for library construction.

**Results:**

High-throughput sequencing produced a total of ~7.4 million high-quality reads, of which approximately 45.07 % were composed of 769 miRNAs and 35.54 % were composed of 11,854 mRNAs target sites. Further bioinformatics analysis identified 43 conserved known miRNAs and 256 novel miRNAs in the SjAgo-associated small RNA population. An average of approximately 15 target sites were predicted for each miRNA. Moreover, a positive rate of 50 % has been achieved in a small-scale verification test of the putative target sites of miRNA1.

**Conclusion:**

In this study, we isolated and identified small RNAs including miRNAs and their targets associated with the *S. japonicum* Argonaute proteins, by the HITS-CLIP method combined with bioinformatics and biologic experimental analysis. These data reveal a genome-wide miRNA-mRNA interaction map in *S. japonicum* in vivo, which will help us understand the complex gene regulatory network in this pathogen and thereby facilitate the development of novel drug approaches against schistosomiasis.

**Electronic supplementary material:**

The online version of this article (doi:10.1186/s13071-015-1203-9) contains supplementary material, which is available to authorized users.

## Background

Human schistosomiasis is one of the most prevalent and serious parasitic diseases in tropical and subtropical regions [1]. It affected approximately 200 million people worldwide. In China, this infectious disease caused by *Schistosoma japonicum* remains a major public health problem [2, 3]. Schistosome parasite undergoes a complex life cycle involving multiple development stages, including egg, miracidium, cercaria, schistosomulum, and adult worm. Each stage may be controlled by various gene regulation mechanisms, which are crucial for development, infection, immune evasion, and pathogenesis of the blood flukes [4]. To date, the genomes of three major pathogenic schistosome species, including that of *S. japonicum,* have been published. However, current understanding of the regulatory mechanisms of stage-specific gene expression is still limited [4–7].

In recent years, microRNAs (miRNAs) have received huge attention as key regulators of gene expression both at transcriptional and post-transcriptional levels in various organisms [8–14]. miRNAs belong to a class of small non-coding RNAs (18–25 nt) generated from endogenous transcripts with hairpin structures [15–19]. Dicer and Argonaute (Ago) are the two core proteins involved in this pathway [20–22]. Primary transcripts of miRNA (pri-miRNA) are transcribed by RNA polymerase II and processed by RNase III in the nucleus. Another RNase III enzyme, Dicer, reprocesses the pri-miRNAs into precursor miRNAs (pre-miRNA). Pre-miRNAs were subsequently transported from nucleus to cytoplasm, where they are sheared into mature miRNA by Dicer. miRNAs bind to the RNA-induced silencing complex (RISC), which contains the Argonaute protein. miRNAs are targeted to the single-stranded complementary mRNA [15, 19, 22]. Recent studies suggested an Ago–miRNA–mRNA ternary complex could be formed, and the technique of high-throughput sequencing of RNAs isolated by crosslinking immunoprecipitation (HITS-CLIP) [23] may allow us to identify the Argonaute-associated miRNAs and their target sites simultaneously.

In schistosomes, the emerging evidence for the existence of miRNAs hinted at the existence of miRNA-mediated gene regulation pathway critical for the gene expression [24–29]. To date, by the conventional polyacrylamide gel electrophoresis (PAGE) enrichment [28], only about 60 miRNAs have been identified for the *Schistosoma* genus, including about 55 in *S. japonicum*, and little is known of their target sites or biological functions [24, 25]. To this end, we aim to systematically analyze the miRNA repertoire and the potential target mRNAs by adapting HITS-CLIP assay [23] in *S.japonicum* with an antibody specific to Argonaute proteins, the core component of RISC complex [30]. Using bioinformatics and molecular biological analysis, researchers have identified and characterized four putative *S. japonicum* Argonaute (SjAgo) orthologues [31]. While the SjAgo2 has been demonstrated to function in maintenance of genome stability via suppression of retrotransposons [26], SjAgo has been speculated to be involved in the miRNA pathway due to its highly conserved functional PIWI and PAZ domains [30]. There are, however, no experimental data available yet.

In the present study, we generated a specific antibody to SjAgo proteins for immunoprecipitation of SjAgo-miRNA-mRNA ternary complex [23]. After enrichment and extraction of the small RNAs associated with the native SjAgo, deep sequencing was carried out on the resulting cDNA library. A total of approximately 7.4 million high-quality reads were produced, and approximately 45.07 % of the reads were composed of miRNAs and 35.54 % were mRNAs. Further bioinformatics analysis identified 43 known miRNAs and 256 novel miRNAs in the SjAgo-associated small RNA population. An average of approximately 15 target sites were predicted for each miRNA. Moreover, partial target sites of miRNA1 were verified by dual luciferase reportor gene assays.

## Methods

### Ethics statement

Animal experiments were performed in accordance with the National Guidelines for the Use and Care of Laboratory Animals, and the study was approved by the Institutional Animal Care and Use Committee of Tongji University.

### Parasites and animals

The *S. japonicum* strain used in this study was obtained from Anhui province of China and was maintained in *Oncomelania hupensis* snails for laboratory studies in China. No permission was required to use parasites from this location in the study. Furthermore, our study did not involve the use of endangered or protected species. To collect *S.japonicum* worms (7d, 14d, 21d, 28d, 35d, and 42d) and eggs, a total of 50 BALB/C mice were infected percutaneously with cercariae of *S. japonicum* (40 worms/mouse) shed from naturally infected snails. No mice died until they were sacrificed for parasite collection. Then, the worms and eggs were collected at desired time points from the mesenteric veins and the livers of the infected mice which were sacrificed by cervical dislocation [24].

### Construction of recombination plasmid

For constructing the recombinant plasmid *pET28a* (+)-*Ago1*-*Piwi*, total RNA of *S.japonicum* was isolated from 28 d adult worms using TRIzol reagent (Invitrogen, USA) and treated with RNase-free DNase. Then, 0.5 μg of total RNA was used as template for the preparation of cDNA [28]. The segment (958 bp) of *SjAgo1* gene was amplified by primers SjAgo1-Piwi-F 5’gccgccatgggatgttcgtttttactacttattct 3’ and SjAgo1-Piwi-R 5’gcgcggatcctcattagtggtgatggtgatggtgaaacatcccatcgacctgcgacata 3’ and was cloned into the expression vector *pET28a* (+) at a multiple cloning site (*Bam*HI and *Nco*I). For protein purification, the coding sequence of six tandem His-tag (His x 6) was fused to the C-terminus of the target sequence [32]. Finally, the cloned sequence was verified using sequencing analysis.

### Expression and purification of the recombinant protein

*pET28a* (+)-*Ago1*-*Piwi* plasmid was transfected into competent BL21 bacteria, and expression of the recombinant protein was induced by 0.1 mM IPTG at 37 °C for 6 h. Protein purification was performed using *E.coli* lysate under denaturing conditions according to the manufacturer’s instructions (Cat: 706663, Novagen). The purity and concentration of the recombinant protein were determined by polyacrylamide gel electrophoresis (PAGE) and Lowry method.

### Preparation of antiserum against SjAgo

Antibodies against recombinant SjAgo were stimulated in New Zealand white rabbits by repeated immunizations. 0.5 mg recombinant protein was briefly emulsified with isopyknic Freund’s complete adjuvant and injected into rabbits subcutaneously. Two booster injections were administered with 500 μg recombinant SjAgo1 in incomplete Freund’s adjuvant at 2-week intervals. Ten days after the final injection, 50 ml of blood was collected and kept overnight at room temperature to allow for clotting of the blood. The crude antiserum was collected by centrifugation at 1500 g for 10 min. The titer and specificity of the antiserum were determined by ELISA and western blotting, respectively.

### Western blotting

Protein samples were mixed with equal volume of 2 × SDS-PAGE loading buffer and boiled at 100 °C for 5 min, then immediately kept it on ice for 10 min. After that protein samples were separated by 10 % SDS-PAGE and transferred to PVDF membrane. Next, the membrane was blocked in 5 % non-fat milk in PBST for 2 h at room temperature. The membrane was then incubated with primary antibody solution (1:2000) for 12 h at 4 °C, followed by reaction with secondary antibody conjugated with HRP for 1 h at room temperature [33]. Finally, the membrane was developed by ECL substrate and detected by GE ImageQuant LAS 4000.

### HITS-CLIP

1. ***Cell lysate*.***S. japonicum fresh adult worms* (~200 mg) were washed 3 times by pre-chilled 1xPBS (pH7.4). To collect single cells, the worms were transferred to a 100 mesh metal sieve and grinded gently with mortar. The cells were suspended with 10 ml pre-chilled 1xPBS (pH 7.4),transferred into a sterile dish with 1 mm depth, and UV-crosslinked using the dose, 400 mJ/cm^2^ for 30 sec each time, twice. After that, the cells were collected by centrifugation at 3000 rpm for 10 min at 4 °C. 700 μl PXL (1xPBS, 0.1 % SDS, 0.5 % deoxycholate, 0.5 % NP-40), protein inhibitors and 15 μl RNasin (Promega) were added and mixed with 1 ml pipette. The mixture was incubated for 10 min on ice. Next, 10 μl RNase (40 U/μl ) was added and incubated for 5 min at 37 °C. Finally, the cell lysate was centrifuged at 14,000 rpm at 4 °C for 20 min, and the supernatant was transferred to a new tube. The cell lysate was stored at −80 °C for co-immunoprecipitation experiment.
2. ***Pretreatment of protein A agarose bead.*** Two milliliters of 0.1 M 1xPBS (pH 8.0) were used to wash protein *A agarose bead* (800 μl) followed by centrifugation at 2000 rpm at 4 °C for 5 min. This step was repeated twice and the protein A*-*agarose were resuspended by 800 μl of 0.1 M 1x PBS (pH 8.0) and divided into two tubes equally. While 50 μl normal serum control was added to tube 1, 50 μl anti-SjAgo serum was added to tube 2. Both tube 1 and 2 were incubated at 4 °C for 4 h. After that, the protein A*-*agarose was collected by centrifugation for 5 min at 2000 rpm at 4 °C.
3. ***Co-Immunoprecipitation.*** 1 ml total protein extracted in step 1 was used to resuspend the pretreated protein A*-*agarose, and the mixture was incubated at 4 °C for 4 h at 100 rpm on a rotator. Then, the protein A*-*agarose was collected by centrifugation for 5 min at 2000 rpm at 4 °C. The supernatant was discarded and the beads were washed 6 times with 1 × PKL buffer. After washing, 200 μl PNK Buffer (50 mM Tris-Cl pH 7.4, 10 mM MgCl_2_, 0.5 % NP-40 ) was added and incubated at 37 °C for 30 min. Then, suspension was obtained by centrifugation at 4 °C at 1400 rpm for 20 min. The RNA-protein complex was analyzed by 8 % SDS-PAGE electrophoresis and transferred to PVDF membrane. The membrane was cut into two parts. Part 1 was used for western blotting to confirm the enrichment of RNA-Ago complex, and part 2 was used to collect RNA-Ago complex for the construction of miRNAs-mRNAs library. After western blotting with SjAgo antiserum, target band in part 2 was cut and washed with DEPC water. Then, 200 μl proteinase-K (4 mg/ml) was added and incubated at 37 °C for 20 min, followed by treatment with 200 μl 1xPKL buffer (100 mM Tris-Cl pH 7.5, 50 mM NaCl, 10 mM EDTA)/7 M urea solution at 37 °C for 20 min. Then, 400 μl phenol and 130 μl chloroform were added to get rid of the protein. After that, 50 μl NaOAc (pH5.2), 0.75 μl glycogen and 1 ml mixture of ethanol and isopropanol (1:1) were added and incubated over night at −20 °C. The next day, the small RNAs were harvested by centrifugation at 14000 g at 4 °C for 30 min. The dried RNA was dissolved in 25 μl DEPC water and was equally divided into two tubes and stored at 80 °C until use. To confirm that the miRNAs and target mRNAs of interest were successfully pulled down by the Co-IP experiment, PCR was performed with known miRNA primers and the PCR products were cloned into T-vector for sequencing analysis. Then, the RNA solution was ligated with Illumina’s proprietary adaptors, and the products were amplified by RT-PCR (TaKaRa, Reverse Transcriptase M-MLV). The purified PCR products were used for clustering and sequencing by an Illumina Genome Analyzer at the Beijing Genomics Institute, Shenzhen. In this section, 200 ng RNA obtained from Co-IP was used for deep sequencing.

### Bioinformatic analysis

All raw data from sequencing were mapped to the *S. japonicum* genome to eliminating the possibility of contamination and to get rid of low quality data. After that, the length distribution of unique sequences was analyzed. To distinguish unique sequences originating from rRNA, tRNA, snRNA (small nuclear RNA) and snoRNA (small nucleolar RNA), the sequences were compared to sequences of non-coding RNAs collected in Rfam 9.0 [34, 35] and the NCBI GenBank data. All rRNAs, tRNAs, snRNAs and snoRNAs were removed before identification of miRNAs and targets sequences [36, 37]. The identification of *S. japonicum* miRNAs and target sites were carried out using previously established criteria [16, 38–40]. Firstly, the precursor sequence of candidate miRNA can form hairpin-like structure. Secondly, the free energy of all predicted hairpin-like structures should be the optimal energy points. Addition to these basic principles, different algorithms make restriction and optimization respectively.

To identify the target sequences of miRNA, RNAHybrid was performed with the predicted miRNAs dataset and the rest of unique data.

### Quantitative RT-PCR of miRNAs expression analysis

RNA was extracted from parasites at various stages by TRIzol method (Invitrogen) according to the manufacturer’s instructions. The residual DNA in the RNA preparations was removed by DNase I treatment. A stem-loop qRT-PCR method [28] was used to measure the expression of individual miRNA. cDNA was synthesized using 1 μg total RNA, 50 nM of each stem-loop RT primer, 0.5 mM dNTP, 5 U M-MLV reverse transcriptase (Takara) and 2 U RNase inhibitor. The program consisted of incubations for 30 min at 16 °C , 30 min at 42 °C, 15 min at 70 °C and then at 4 °C indefinitely. Real-time quantification was performed using the Applied Biosystems 7300 Sequence Detection System. The 20 μl PCR reaction included 2 μl of cDNA (10:1dilution), SYBR Premix Ex Taq II (Takara), 0.5 mM specific forward primer and 0.5 mM common reverse primer. The reactions were run at 95 °C for 10 s, followed by 40 cycles of 95 °C for 5 s and 60 °C for 31 s. The 2^-△△Ct^ method was used to calculate the relative copy numbers of each miRNA with U6 RNA as the internal standard.

### Cell culture and luciferase reportor assays

HEK293T cells were cultured as described previously [41]. Luciferase reportor assays were developed following the protocol of the p^mir^GLO Dual-Luciferase miRNA Target Expression Vector Report System (Cat: E1330 Promega). Briefly, miRNA duplexes and 2’-O-methyl oligonucleotides mimics (miR-1, miR-21) were chemically synthesized by Genepharma (Shanghai, China) and used for the transfection of HEK293T cells after in-vitro annealing. Meanwhile, miRNA target sequences and control sequences were synthesized and cloned into the p^mir^GLO vector through multiple cloning sites located in the downstream of luciferase (firefly luciferase/renilla luciferase) gene. Finally, recombinant plasmids were sequenced to confirm that the target sequences were cloned correctly. One hundred and fifty nanograms of recombinant plasmid and 160 ng miRNA mimic were co-transfected by X-trene GENE Transfection Reagent (Roche) in 96-well plates. Luciferase activity was measured after 24 h using the dual luciferase assay system (Cat: E1500 Promega).

## Results

### Generation of anti-SjAgo antibody for HITS-CLIP

**Fig. 1:**
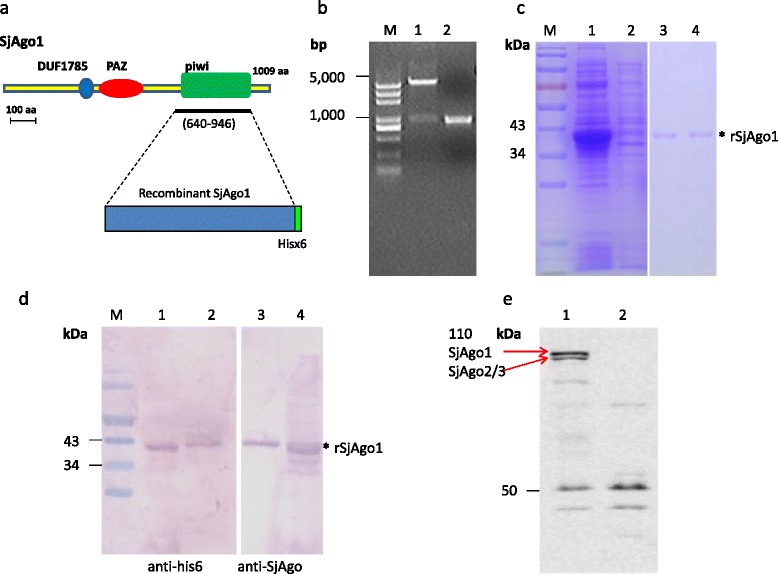
Generation of the anti-SjAgo1 antibody. **a** Schematic representation of the full-length SjAgo1 protein and the recombinant rSjAgo1 protein with Hisx6 at the C-terminus as an affinity purification tag. **b** Restriction enzyme digestion analysis of the recombinant plasmid *pET28a* (+)-*Ago1*-*Piwi*. Lane 1 is the recombinant plasmid digested with *BamH* I and *Nco* I; lane 2 is the control, consisting of the cloned fragment of the *SjAgo1* gene. M. DNA ladder. **c** Coomassie brilliant blue staining of a 12 % SDS-PAGE gel of the total *E.coli* lysate (1), flow-through (2), and eluted fractions (3 and 4) from the purification of rSjAgo1 by nickel column chromatography. M. prestained protein ladder. **d** Western blot assay of rSjAgo1. Lane1 and lane 3 are purified rSjAgo1; Lane 2 and lane 4 are crude *E.coli* lysate. M. prestained protein ladder. **e** Western blot assay of total extract of *S.japonicum* with anti-SjAgo serum (1) or pre-immune serum (2). The recombinant rSjAgo1 is indicated by an asterisk

A growing body of evidence suggests that SjAgo plays an important role in the miRNA pathway [20, 22] and SjAgo1 is the most closely related orthologue to the ancestral Argonaute known to bind miRNA in other organisms based on phylogenetic analysis [31]. To investigate the native SjAgo-associated miRNAs and their potential targets through the HITS-CLIP assay, a specific antibody against SjAgo was produced in the present study. Due to the relatively large molecular mass of SjAgo1 protein (~109 kDa) which may lead to difficulty in expressing the whole SjAgo1 protein in prokaryotic expression system, truncation expression was performed i.e., a fragment of the *SjAgo1* gene (aa 640–946) covering the conserved PIWI domain among SjAgo1/2/3 was amplified and cloned into the vector, *pET28a* (+) (Fig. 1a). A double enzyme digestion was conducted to confirm that the target sequence had been inserted. Furthermore, sequencing analysis of the inserted gene fragment in the recombinant plasmid was performed (Fig. 1b). Next, expression of the recombinant protein rSjAgo was induced by 0.1 mM IPTG in the BL21 bacterial strain and purified by affinity purification (Fig. 1c). Rabbits were subcutaneously immunized with the purified recombinant protein to generate specific antibody to SjAgo. ELISA and western blotting experiments showed that the antiserum had high titer and specificity. The antibody was able to recognize both recombinant SjAgo1 protein and full-length native SjAgos including Ago1, Ago2, or Ago3 in total cell lysate of *S.japonicum* (Fig. 1d and e).

### UV cross-linking and co-immunoprecipitation

**Fig. 2:**
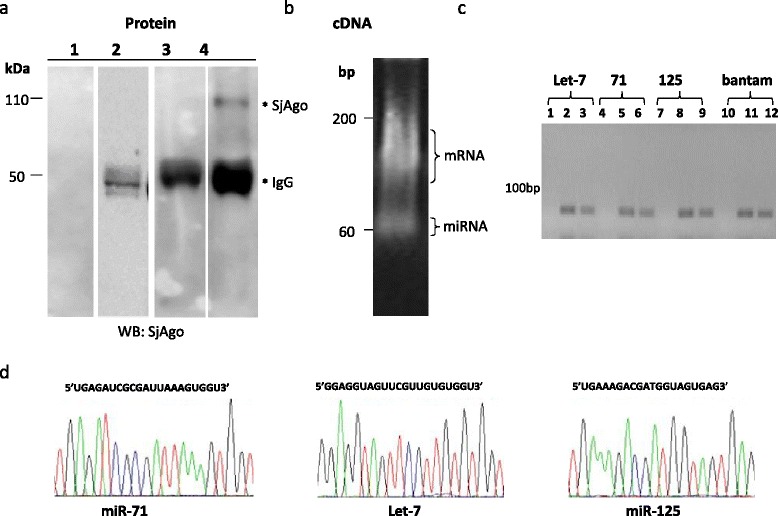
Immunoprecipitation of native SjAgo-miRNA-mRNA complex with anti-SjAgo1 antibody. **a** Western blot assay of immunoprecipitated SjAgo complex. Lane 1 (control): total parasite extract was directly incubated with Protein A agarose without immune serum; Lane 2 (control): pre-immune serum; Lane 3 (control): only immune serum against SjAgo was incubated with Protein A agarose without total parasite extract; Lane4: Protein A-agarose was incubated with immune serum against SjAgo in the presence of total parasite extract. The SjAgo complex and IgG are indicated by asterisks. **b** RT-PCR products of the small RNAs isolated from the RNA-protein complex after ligation of linkers (17 + 17 nt). cDNA was synthesized according to the manufacturer’s instructions (TaKaRa), then PCR was performed (35 cycles). The putative miRNA and mRNA populations were indicated. **c** and **d** Verification of four previously identified *S.japonicum* miRNAs by PCR analysis. **c** Lanes 1, 4, 7, 10 are the negative control, lanes 2, 5, 8, 11 are the positive control, and lanes 3, 6, 9, 12 are the experimental groups of cDNAs from Fig. 2b. **d** Sequencing of the PCR products amplified from the cDNAs from the SjAgo complex

To enhance the stability of SjAgo-miRNA-target complex, UV cross-linking was performed before extraction of total protein. For enrichment of the SjAgo-miRNA-target complex from total protein, co-immunoprecipitation [42] was adapted in our study. As shown in Fig. 2a, SjAgo was successfully pulled down by co-immunoprecipitation using anti-SjAgo serum. To avoid false positive reactions, three control experiments were performed simultaneously. As expected, no signal was detected in corresponding positions in western blots with pre-immune serum controls. However, we do not know whether the miRNAs and their target sites were enriched simultaneously with the native SjAgo in this experiment. The products obtained from co-immunoprecipitation were used for RNA extraction. Then, RT-PCR was performed using stem-loop primers, which were designed according to a few representative known miRNAs of *S.japonicum* (see Additional file 1). As shown in Fig. 2b-d, native SjAgo-associated miRNAs and potential target mRNAs were successfully enriched and isolated through the co-immunoprecipitation experiment. Taken together, these results showed that the SjAgo-miRNA-target complex can be successfully isolated via UV cross-linking immunoprecipitation with the specific SjAgo antibody.

### High-throughput sequencing and data analysis

**Fig. 3:**
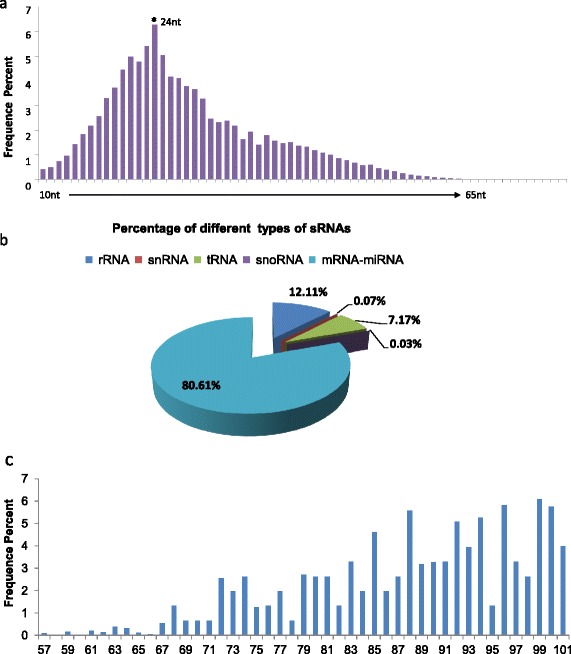
Length range and proportion of the small RNAs isolated from the native SjAgo complex. **a** The isolated small RNAs with a length range from 10 to 65 nt. **b** The proportion of various small RNAs determined by the HITS-CLIP assay. **c** The isolated mRNA with a length range from 57 to 101 nt

Overall, a total of 7,428,407 reads of small RNAs were generated by SOLEXA sequencing including 596,991 unique contigs. The length of small RNAs ranged from 10 to 65 nt with a peak at 24 nt (Fig. 3a). Further analysis showed that out of the total small RNA sequences, 899,947 (12 %) come from ribosomal RNA (rRNA), 1,926 (0.025 %) from Small nucleolar RNA (snoRNA), 532,839 (7 %) from transfer RNA (tRNA), 5,427 (0.07 %) from small nuclear RNA (snRNA), 3,347,983 (45.07 %) from miRNAs, and 2,640,056 (35.54 %) from mRNAs (Fig. 3b). The average length of mRNAs in these datasets are 88 nt (Fig. 3c). Next, the sequences of potential miRNAs and mRNAs were clustered as a data set and used to identify reliable miRNAs and their target sites. As shown in Table 1 and Additional file 2, compared to Sanger miRNA database (http://www.mirbase.org/), 513 putative known miRNAs which have been identified and released in miRNA-database in all species in previous study were detected, of which 43 conserved miRNAs such as miR-1, miR-21, let-7, etc. had been identified in *S.japonicum* previously [22, 26, 32]. Interestingly, 256 novel miRNAs (Table 1 and Additional file 3) containing perfect precursor secondary structure with optimal folding free energy (see Additional file 4) had been found to be associated with the native SjAgo. To identify the target sequences of these miRNAs, RNAHybrid prediction was performed with the predicted miRNAs data set and the rest of unique data [43]. Any sequence containing a region that can match to the seed sequence of a given miRNA with the lower free energy was considered to be a candidate target site for this miRNA [38, 44]. The results showed that a total of 7414 unique sequences were matched to the “seed” sequences of the 513 putative known miRNAs, and there were 4440 potential target sites for the 256 novel miRNAs (see Additional files 5 and 6). This indicates an average of 15 target sites matched to each native SjAgo-associated miRNA. Thus, this study was able to establish the SjAgo-associated miRNA-mRNA interaction network. Nevertheless, all of these miRNA-mRNA pairings need to be verified by biological experiments such as luciferase reportor assays [8, 45].

**Table 1:** Analysis of microRNAs and targets found in adult S.japonicum by SOLEXA deep sequencing

Item	Putative miRNAs	Analysis results**	Total targets	Average number of targets per miRNA
Known miRNAs	513*	43	7414	~15
Novel miRNAs	256	256	4440	~17

*putative miRNAs found in miRBase (http://www.mirbase.org/)

**further bioinformatics analysis of those putative miRNAs with interspecific or intraspecific sequence conservation, precursor secondary structure with optimum folding free energy, higher abundance, etc

### Luciferase reportor assay to validate the potential target sites

**Fig. 4:**
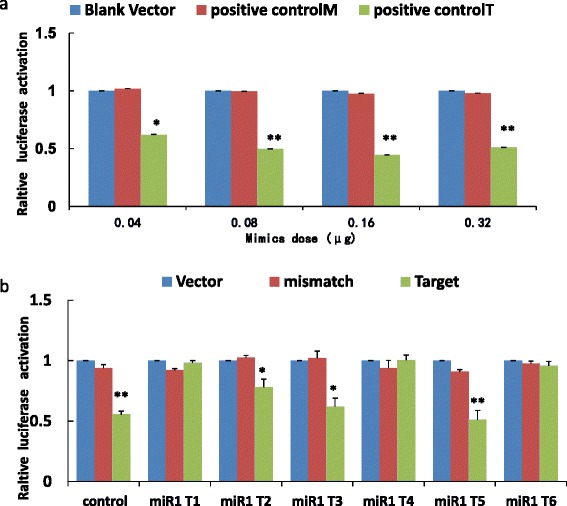
Verification of the target sites of S.japonicum miRNAs by luciferase reporter assays. **a** Positive control with different mimic doses suppressed the activity of the target site. **b** Verification of partial putative target sites of known miRNA1 found by HITS-CLIP. Data are presented as the mean ± SD of triplicate independent experiments. “*”: *p* < 0.05; “**”: *p* < 0.001 (Student’s *t*-test)

Firstly, we optimized the transfection conditions for HEK293T cells to yield a maximum incorporation efficiency of miRNAs (see Fig. 4a and Additional file 7: Table S1). To validate the potential target sites, 6 target sites of miR-1 were randomly selected and cloned into p^mir^GLO vector through MCS located in the 3’UTR of the luciferase gene. The examined sequences of target sites and their corresponding mismatch sites were shown in Additional file 7: Table S2. The mismatch sequence was mutated in the target site which matched to the seed sequence of miRNA-1. Then, miRNA mimics and recombinant plasmids containing potential target sites were co-transfected into HEK293T cells to investigate whether the target site was really interacted with the miRNA. An interaction can lead to reduction in the expression of the firefly luciferase gene. As shown in Fig. 4 and Table 2, the activity of firefly luciferase was significantly reduced in 3 out of 6 miRNA1 groups, indicating that there are multiple target sites of individual miRNAs. In addition, these data provide a compatible efficiency (50 %) of target prediction for *S.japonicum* miRNAs by the HITS-CLIP method developed in this study.

**Table 2:** miRNA targets identified by the dual-luciferase expression vector reportor system

miRNA	Target site	ID number	Validated
miRNA1	T1	T0054493	NO
miRNA1	T2	T0198367	YES
miRNA1	T3	T0090332	YES
miRNA1	T4	T0196767	NO
miRNA1	T5	T0023779	YES
miRNA1	T6	T0149441	NO

## Discussion

HITS-CLIP had been successfully used in the identification of native Argonaute-associated miRNAs and their target sites in mouse brain previously [23]. In that study, the investigators found the positive rate of predictive targets by Ago-miR-124 was up to 73 % with 92.5 % specificity. This level of specificity is much higher than the specificity achieved by using an analysis of conserved seed sequences alone. In the present study, approximately 81 % of small RNAs immunoprecipitated from the SjAgo1-miRNA complex were miRNAs and mRNAs. miRNAs are recognized as important regulators of gene expression through specific base-paring with their target mRNAs [10, 18, 46]. A bioinformatic approach, incorporating sequence matching and mRNA secondary structure to predict mRNA targets [36, 47, 48], revealed multiple highly conserved binding sites for miRNAs in the 3’ untranslated region (UTR) [16, 37]. However, luciferase dual-reportor detection indicated that, as reported by Sung’s study, some of the miRNAs targets predicted by bioinformatics were false positives [49–51].

miRNAs, like transcription factors, are an abundant class of gene-regulatory molecules in animal cells. miRNAs regulate a variety of developmental, differentiation and physiological processes [6, 15, 17, 52]. Most miRNAs are expressed in a development- or organ-specific manner, which provide hints about their functions [8, 9]. It was observed that Ago is essential for mature miRNA production, thereby it is the primary factor responsible for the small RNA silencing pathway. Luo et al. analyzed the expression of SjAgo in different developmental stages of *S. japonicum* [31]. The expression pattern of SjAgo during the life cycle of *S. japonicum* indicates that the miRNA regulatory pathway might take part in the transformation and development of *S. japonicum*. However, functional specificities associated with different Argonautes remain elusive. Wang et al. have reported that

microRNAs are randomly sorted to individual Argonautes in mammals [53]. In the platyhelminth *S. japonicum*, miRNAs may take part in a regulatory network along with transcription factors and growth factors to control the development and differentiation of the parasites [6]. Profiling of miRNA1, miRNA21, miRNAlet-7 expression by stem-loop quantitative RT-PCR revealed highly stage-specific expression patterns (see Additional file 8). Importantly, the expression peaks of miRNA1 appeared in schistosomulum, the stage after infection of the host. These findings indicate that these miRNAs may be involved in schistosome growth and development.

The miRNA-71 family is a conserved microRNA cluster in both *S. japonicum* and *S.mansoni.* Xue et al. cloned the miRNA-71a cluster in adult worm and analyzed their expression level across the lifespan of *S.japonicum* [24, 28]. Subsequently, the same group demonstrated that miRNA-71a cluster and miRNA-71b cluster were expressed in schistosomulum, using deep sequencing and northern blotting. However, in the present study, only miR71c was found to be associated with native SjAgo1 by HITS-CLIP analysis. There is a possibility that the third *S.japonicum* Argonaute protein, SjAgo3, may also participate in the processing of miRNA in a complementary pathway, because both SjAgo1 and SjAgo3 molecules contain PIWI and PAZ domains [30]. A further HIST-CLIP assay with antibodies specific to SjAgo3 protein would give us more information on the different Argonaute-dependent miRNA pathways controlling gene regulation in various physiological processes in *S. japonicum*.

Controlling schistosomiasis remains a huge challenge in endemic areas, particularly in developing countries [2, 3], due to little knowledge on *S. japonicum* physiology. Considering the distinct developmental stages in vertebrate and invertebrate hosts and the unique repertoire of genes expressed at different life cycle stages of the schistosome parasites [6], exploring the regulatory role of miRNAs in gene expression involved in the parasite’s development, differentiation, and ability to infect mammalian hosts [4, 6, 54] will be particularly important for understanding the mechanistic details of its physiology and pathogenesis. To this end, a systematical identification and characterization of *S.japonicum* miRNAs and their target sites by those technique such as Argonaute-associated HITS-CLIP [23] is the first and essential step towards understanding the biological regulatory functions and related mechanisms of miRNAs in blood flukes [11]. Such identification may enable the understanding of the biological basis of growth, antigenic diversity, infection, and pathogenesis of the flukes and thereby contribute to the development of new approaches to prevent and treat schistosomiasis by providing novel drug targets and vaccine candidates.

## Conclusions

*Schistosomes* are the responsible pathogens for schistosomiasis, which is one of the most prevalent, but neglected, parasitic diseases in tropical and subtropical countries. Although some *S. japonicum* miRNAs have been discovered in the past decade, a systematic analysis of miRNAs and their target sites in this parasite has not been carried out. In the present study, we have adapted the HITS-CLIP technique to investigate the native *S.japonicum* Argonaute proteins (SjAgo)-associated miRNAs and their target mRNAs by generating a specific antibody against SjAgo. Our data revealed a total of 769 miRNAs and 11,854 potential target sites, including 43 known *S.japonicum* miRNAs. We demonstrated the efficiency of target prediction of *S.japonicum* miRNAs by biological verification tests. This study not only expands the miRNA database of *S.japonicum* but also establishes a SjAgo-associated miRNA-mRNA interactome, which advances our understanding of the regulatory network of gene expression in this pathogen.

## Additional files

Additional file 1:
**Primer sequences used in qPCR assays of S.japonicum miRNAs.** (DOC 33 kb) Additional file 2:
**Sequences of SjAgo-associated putative known miRNAs by the HITS-CLIP assay.** (XLS 69 kb) Additional file 3: Sequences of SjAgo-associated novel miRNAs by the HITS-CLIP assay. (XLSX 16 kb) Additional file 4:
**Perfect precursor secondary structure of SjAgo-associated novel miRNAs.** 256 novel miRNAs containing perfect precursor secondary structure with optimal folding free energy using RNA fold software. (PDF 1173 kb) Additional file 5:
**Sequences of SjAgo-associated known miRNAs and targets detected by the HITS-CLIP assay.** (XLS 137 kb) Additional file 6:
**Sequences of SjAgo-associated novel miRNAs and targets detected by the HITS-CLIP assay.** (XLS 1342 kb) Additional file 7:
**Optimized transfection conditions for miRNA transfection into HEK293T cells and Sequence alignment of target site examined and corresponding mutated site. **(PDF 264 kb) Additional file 8:
**Expression levels of three representative miRNAs across the life cycle of S. japonicum.** The expression levels of three miRNAs were normalized to the expression levels of endogenous U6. The data represent the mean ± SD for triplicate independent experiments. (PDF 100 kb)
