# Supplementary material for: High-throughput sequencing of RNAs isolated by cross-linking immunoprecipitation (HITS-CLIP) reveals Argonaute-associated microRNAs and targets in Schistosoma japonicum
Source: Parasit Vectors. 2015 Nov 14;8:589. doi: 10.1186/s13071-015-1203-9 (PMC4650335; doi:10.1186/s13071-015-1203-9)
Supplement: Additional file 1: — Primer sequences used in qPCR assays of S.japonicum miRNAs. (DOC 33 kb) [file 13071_2015_1203_MOESM1_ESM.doc]

**Additional file 1 - Primer sequences used in qPCR assay of *S.japonicum* miRNAs.**

| **miRNA Primer sequence** | | |
| --- | --- | --- |
| **Sj-let-7** | RT Stem-loop Primer: | CTCAACTGGTGTCGTGGAGTCGGCAATTCAGTTGAGAAACTATA |
|  | Forward Primer: | ACACTCCAGCTGGGGGAGGTAGTTCGTTG |
| **Sj-1** | RT Stem-loop Primer: | CTCAACTGGTGTCGTGGAGTCGGCAATTCAGTTGAGACCATACT |
|  | Forward Primer: | ACACTCCAGCTGGGCCCCTGAGACCCTAA |
| **Sj-21**  **Sj-U6** | RT Stem-loop Primer: | CTCAACTGGTGTCGTGGAGTCGGCAATTCAGTTGAGAGATCAAC |
|  | Forward Primer:  RT Primer  Forward Primer  Reverse Primer | ACACTCCAGCTGGGTGAGATCGCGATTAAA  TATGGAACGCTTCACGATTTTG  CGGCGGTACATATACTAAAAT  AACGCTTCACGATTTTGCGT |
| Common reverse Primer: | | CTGGTGTCGTGGAGTCGGCAA |
