# Supplementary material for: High-throughput sequencing of RNAs isolated by cross-linking immunoprecipitation (HITS-CLIP) reveals Argonaute-associated microRNAs and targets in Schistosoma japonicum
Source: Parasit Vectors. 2015 Nov 14;8:589. doi: 10.1186/s13071-015-1203-9 (PMC4650335; doi:10.1186/s13071-015-1203-9)
Supplement: Additional file 4: — Perfect precursor secondary structure of SjAgo-associated novel miRNAs. 256 novel miRNAs containing perfect precursor secondary structure with optimal folding free energy using RNA fold software. (PDF 1173 kb) [file 13071_2015_1203_MOESM4_ESM.pdf]

Precursor secondary structure of SjAgo-associated novel miRNAs

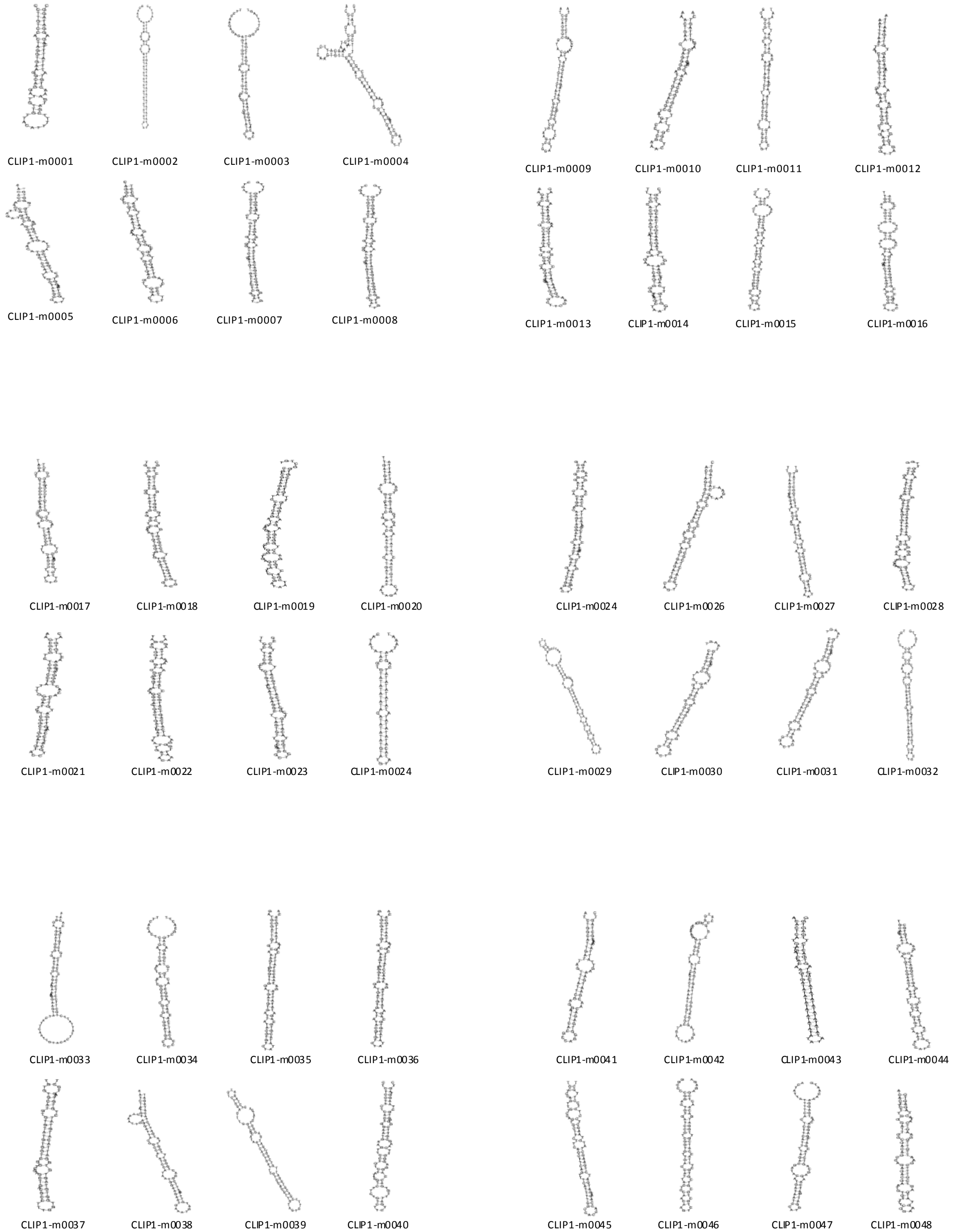

Precursor secondary structure of SjAgo-associated novel miRNAs

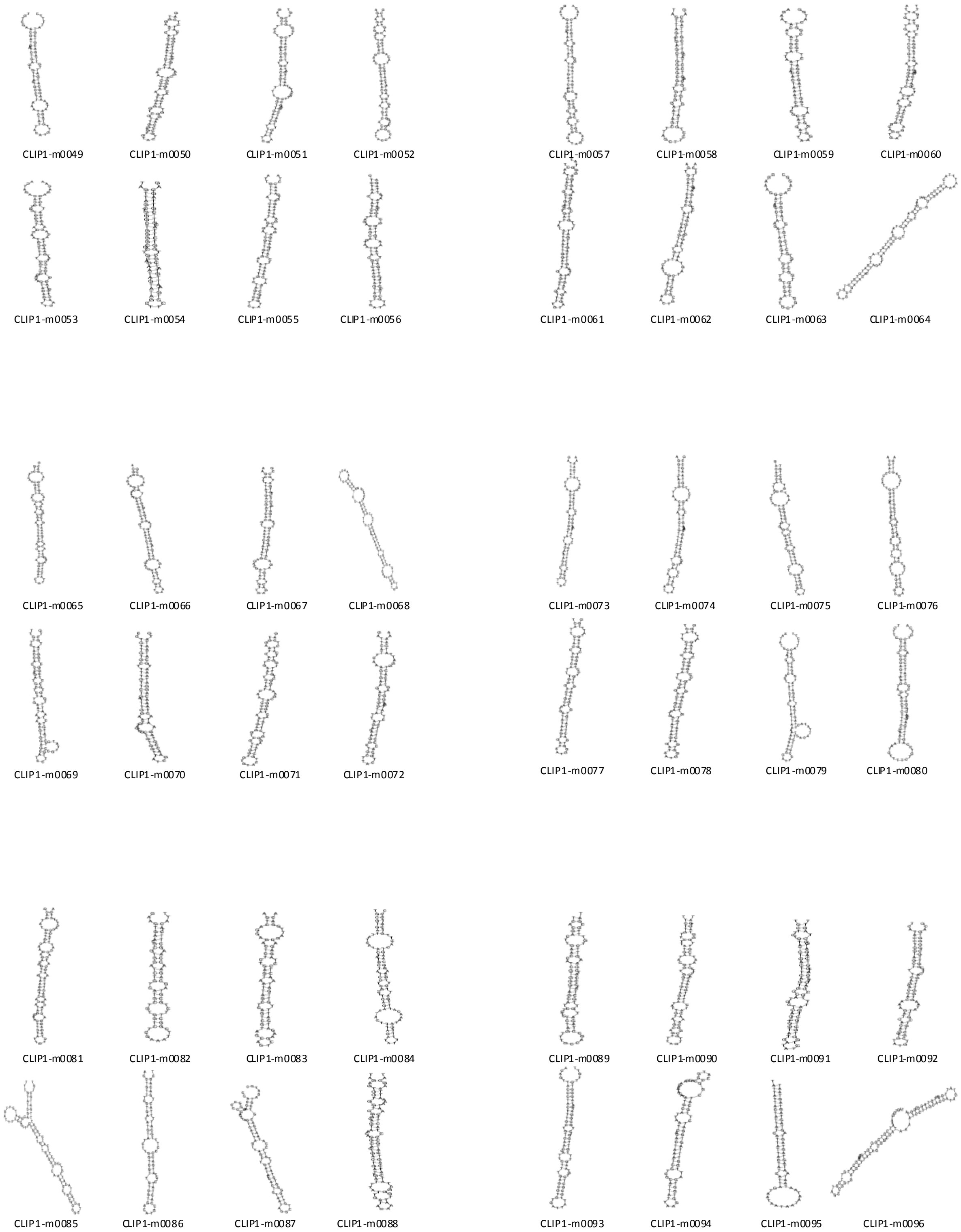

Precursor secondary structure of SjAgo-associated novel miRNAs

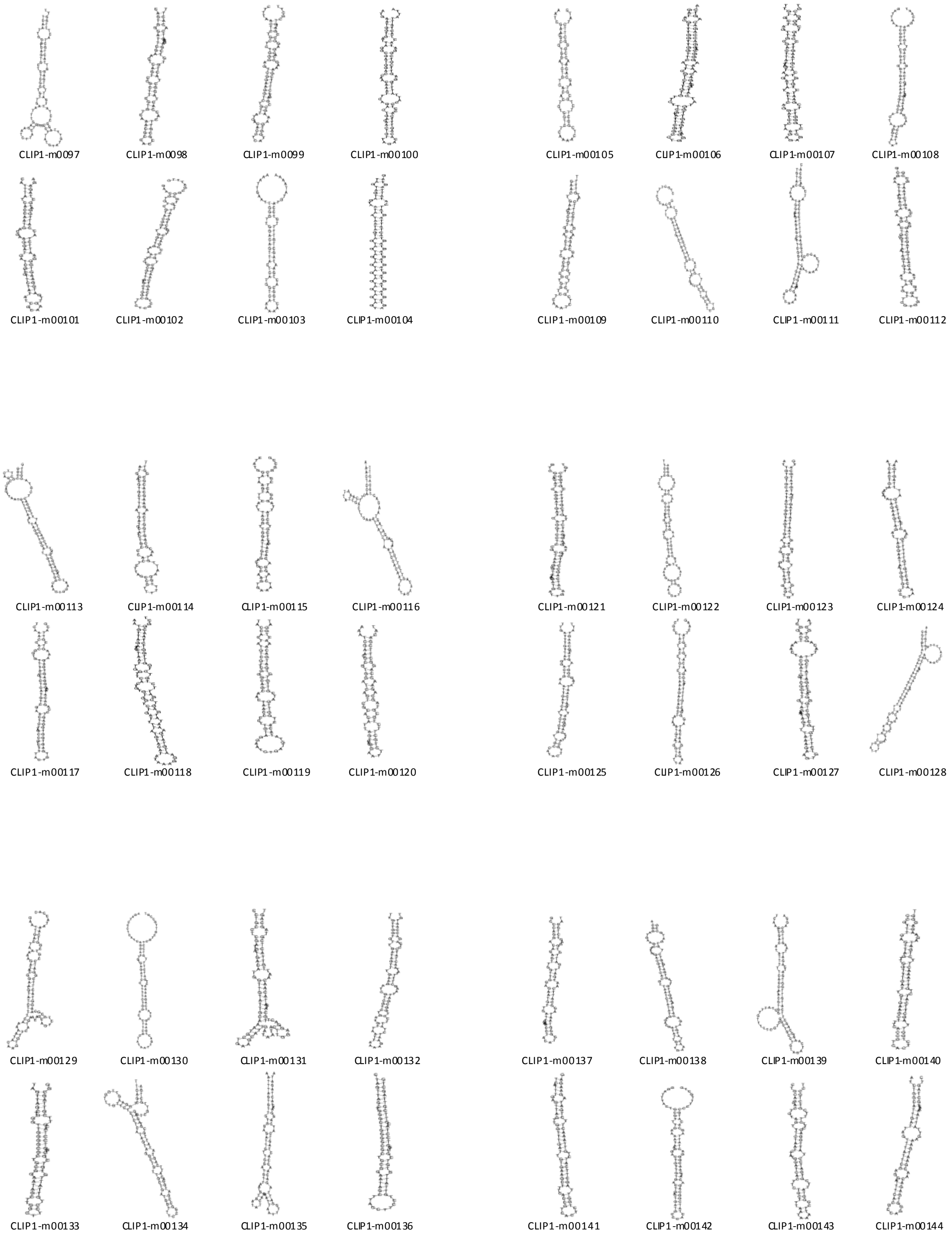

Precursor secondary structure of SjAgo-associated novel miRNAs

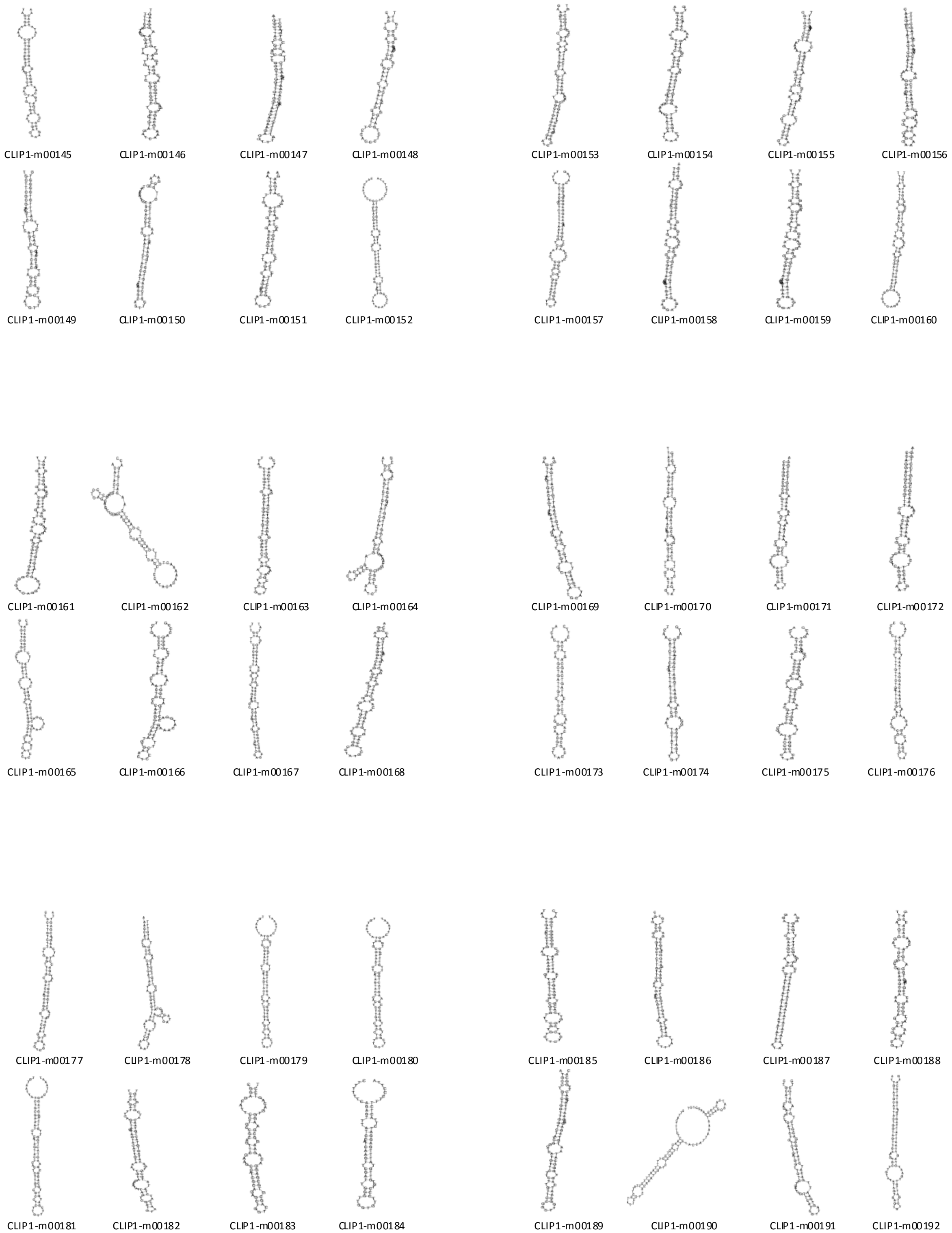

Precursor secondary structure of SjAgo-associated novel miRNAs

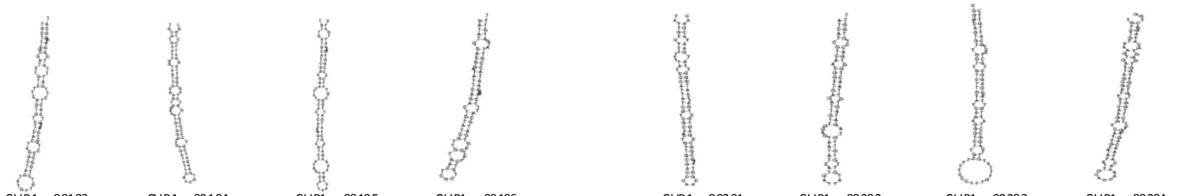

CLIP1-m00193 CLIP1-m00194 CLIP1-m00195 CLIP1-m00196 CLIP1-m00201 CLIP1-m00202 CLIP1-m00203 CLIP1-m00204

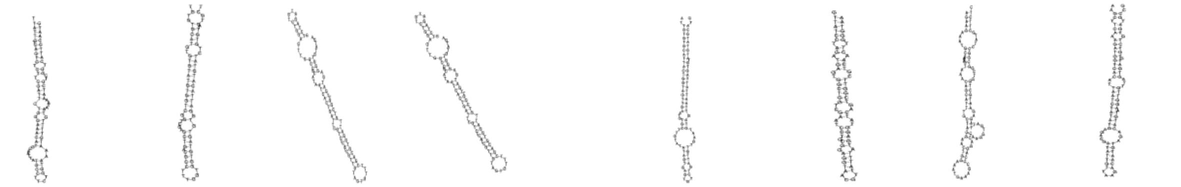

CLIP1-m00197 CLIP1-m00198 CLIP1-m00199 CLIP1-m00200 CLIP1-m00205 CLIP1-m00206 CLIP1-m00207 CLIP1-m00208

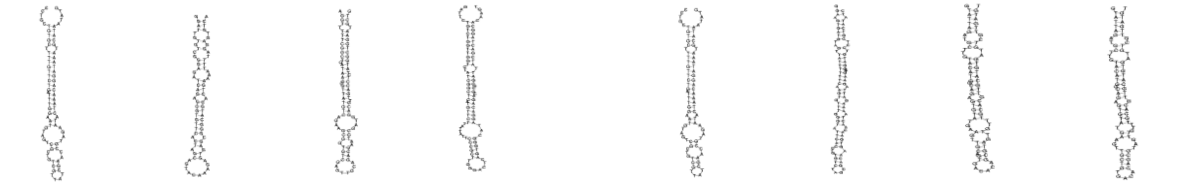

CLIP1-m00209 CLIP1-m00210 CLIP1-m00211 CLIP1-m00212 CLIP1-m00217 CLIP1-m00218 CLIP1-m00219 CLIP1-m00220

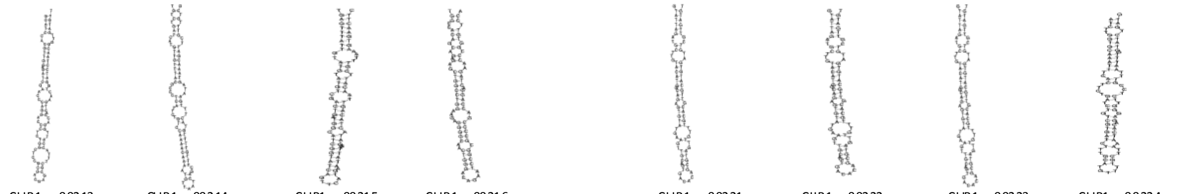

CLIP1-m00213 CLIP1-m00214 CLIP1-m00215 CLIP1-m00216 CLIP1-m00221 CLIP1-m00222 CLIP1-m00223 CLIP1-m00224

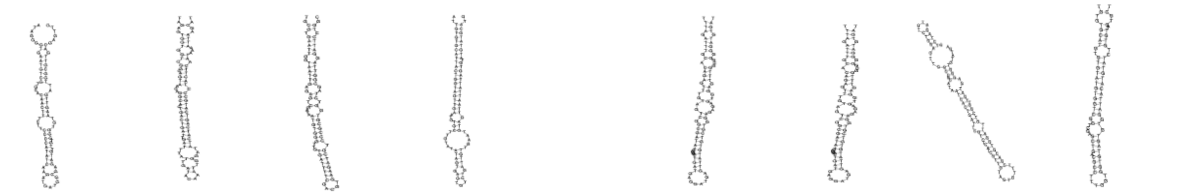

CLIP1-m00225 CLIP1-m00226 CLIP1-m00227 CLIP1-m00228 CLIP1-m00233 CLIP1-m00234 CLIP1-m00235 CLIP1-m00236

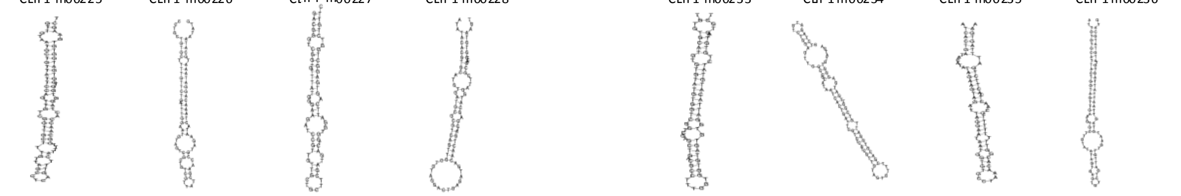

CLIP1-m00229 CLIP1-m00230 CLIP1-m00231 CLIP1-m00232 CLIP1-m00237 CLIP1-m00238 CLIP1-m00239 CLIP1-m00240

Precursor secondary structure of SjAgo-associated novel miRNAs

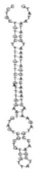
CLIP1-m00241

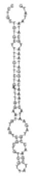
CLIP1-m00242

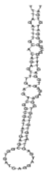
CLIP1-m00243

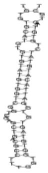
CLIP1-m00244

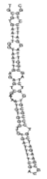
CLIP1-m00249

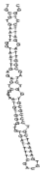
CLIP1-m00250

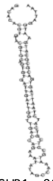
CLIP1-m00251

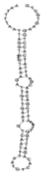
CLIP1-m00252

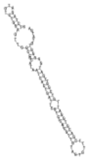
CLIP1-m00245

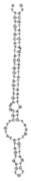
CLIP1-m00246

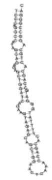
CLIP1-m00247

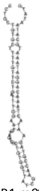
CLIP1-m00248

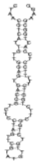
CLIP1-m00253

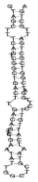
CLIP1-m00254

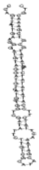
CLIP1-m00255

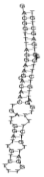
CLIP1-m00256
