# Supplementary material for: High-throughput sequencing of RNAs isolated by cross-linking immunoprecipitation (HITS-CLIP) reveals Argonaute-associated microRNAs and targets in Schistosoma japonicum
Source: Parasit Vectors. 2015 Nov 14;8:589. doi: 10.1186/s13071-015-1203-9 (PMC4650335; doi:10.1186/s13071-015-1203-9)
Supplement: Additional file 7: — Optimized transfection conditions for miRNA transfection into HEK293T cells and Sequence alignment of target site examined and corresponding mutated site. (PDF 264 kb) [file 13071_2015_1203_MOESM7_ESM.pdf]

Additional file 7: Table S1: Optimized transfection conditions for miRNA transfected into HEK293T cells

**Culture plate diameter [mm]**	**96-well plate**
Surface area cm^2	0.4
Plated cells [$*10^5$]	0.15
Medium volume [ml]	0.15
miRNA mimics [µg]	0.16
Vector [µg]	0.15
XtremeGENE Transfection Reagent [µl]	0.12
Dilution volume [µl]	15
Total volume [ml]	0.18

Additional file 7: Table S2: Sequence alignment of target site examined and corresponding mutated site

```
             1                                                      55
miRNA-1 MT1  CACATACTTCTGTATGCTGTAAGCTAGCGTACATACTTCTGTATGCTGTAAGCTT
miRNA-1 WT1  CACATACTTCTGTATGCACATTTCTAGCGTACATACTTCTGTATGCACATTTCTT

             1                                                    53
miRNA-1 MT2  CACTATACTTCAATTATGTAAGTGTGCGAACTATACTTCAATTATGTAAGTGT
miRNA-1 WT2  CACTATACTTCAATTAATATTCTGTGCGAACTATACTTCAATTAATATTCTGT

             1                                              47
miRNA-1 MT3  CACATGCTTTAATTGTAAGCTAGCGTACATGCTTTAATTGTAAGCTT
miRNA-1 WT3  CACATGCTTTAATATATTTCTAGCGTACATGCTTTAATATATTTCTT

             1                                                          61
miRNA-1 MT4  CATCATGCTTCATTTATTGATGTAAGACTGCGAATCATGCTTCATTTATTGATGTAAGACT
miRNA-1 WT4  CATCATGCTTCATTTATTGACATTTCACTGCGAATCATGCTTCATTTATTGACATTTCACT

             1                                                            63
miRNA-1 MT5  ATCATGCTTCATTTATTGACATGTAAGACTGCGAATCATGCTTCATTTATTGACATGTAAGAC
miRNA-1 WT5  ATCATGCTTCATTTATTGACACATTTCACTGCGAATCATGCTTCATTTATTGACACATTTCAC

             1                                                  51
miRNA-1 MT6  ATATACTTCGTTTCATGTAAGAGTGCGAATATACTTCGTTTCATGTAAGAG
miRNA-1 WT6  ATATACTTCGTTTCACGTTTCAGTGCGAATATACTTCGTTTCACGTTTCAG
```
