# Supplementary figures and images for: High-throughput sequencing of RNAs isolated by cross-linking immunoprecipitation (HITS-CLIP) reveals Argonaute-associated microRNAs and targets in Schistosoma japonicum
Source: Parasit Vectors. 2015 Nov 14;8:589. doi: 10.1186/s13071-015-1203-9 (PMC4650335; doi:10.1186/s13071-015-1203-9)

## Differential expression of miRNAs across the life cycle of *S. japonicum*

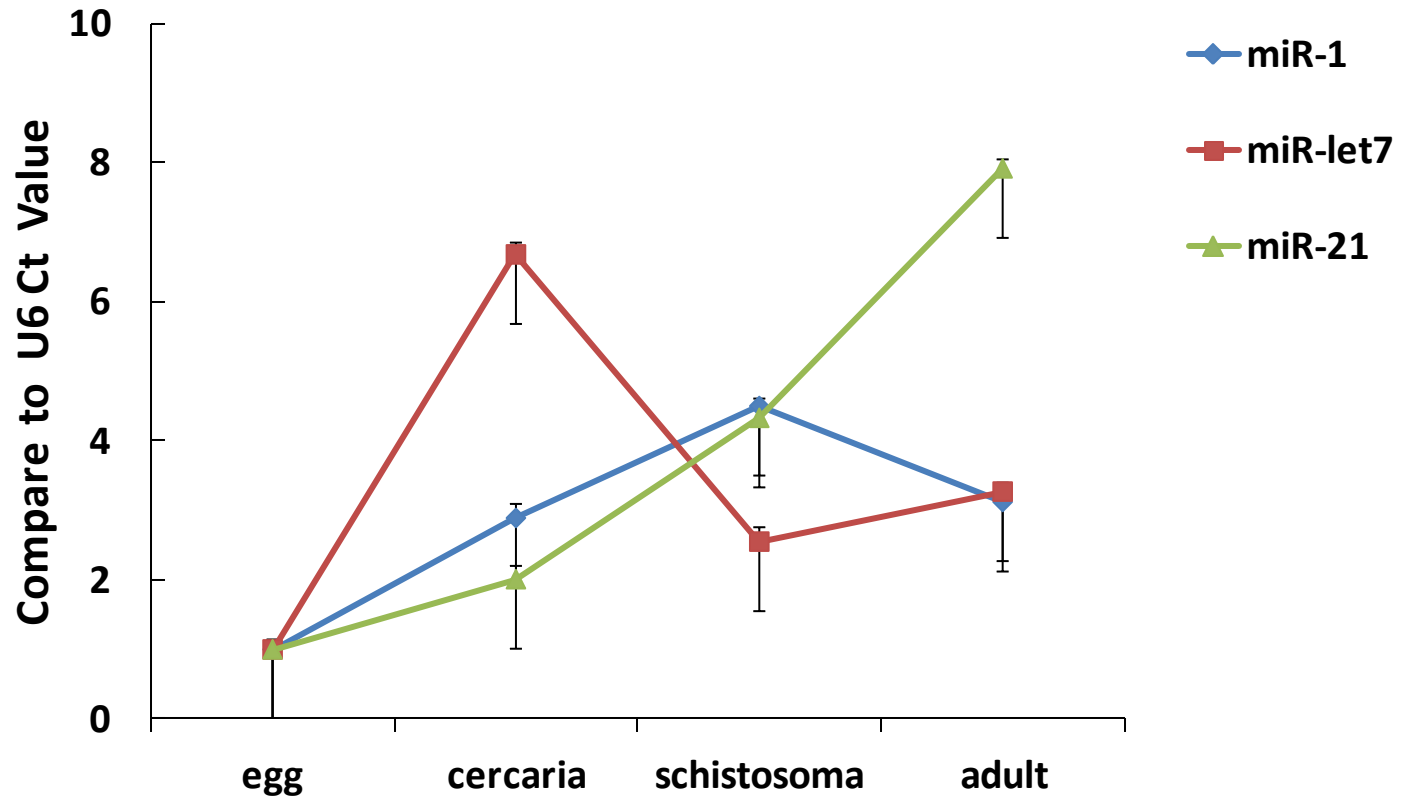

Supplement: Additional file 8: — Expression levels of three representative miRNAs across the life cycle of S. japonicum. The expression levels of three miRNAs were normalized to the expression levels of endogenous U6. The data represent the mean ± SD for triplicate independent experiments. (PDF 100 kb) [file 13071_2015_1203_MOESM8_ESM.pdf]
